# Natural Killer Cell Line NK-92-Mediated Damage of Medically Important Fungi

**DOI:** 10.3390/jof7020144

**Published:** 2021-02-17

**Authors:** Stanislaw Schmidt, Marie Luckowitsch, Michael Hogardt, Thomas Lehrnbecher

**Affiliations:** 1 Division of Pediatric Hematology and Oncology, Hospital for Children and Adolescents, University Hospital Frankfurt, Goethe University, 60590 Frankfurt am Main, Germany; Marie.Luckowitsch@kgu.de (M.L.); Thomas.Lehrnbecher@kgu.de (T.L.); 2 Institute for Medical Microbiology and Infection Control, University Hospital Frankfurt, Goethe University, 60590 Frankfurt am Main, Germany; Michael.Hogardt@kgu.de

**Keywords:** natural killer cell, NK-92, antifungal activity, *Aspergillus*, *Candida*, *Fusarium*, mucormycetes

## Abstract

Invasive fungal disease (IFD) in hematopoietic stem cell transplantation is associated with high morbidity and mortality. As the antifungal host response determines risk and outcome of IFD, there is growing interest in adoptive immunotherapy using T cells or natural killer (NK) cells. Although the NK-92 cell line has been tested as anticancer therapy in clinical trials, data on the antifungal activity of NK-92 cells are lacking. Here, we show that the NK-92 cell line exhibits considerable fungal damage on all medically important fungi tested, such as different species of *Aspergillus*, *Candida*, mucormycetes, and *Fusarium*. The extent of fungal damage differs across various species of mucormycetes and *Fusarium*, whereas it is comparable across different species of *Aspergillus* and *Candida*. Interferon (IFN)-γ levels in the supernatant were lower when NK-92 cells are co-incubated with *Aspergillus fumigatus*, *Candida albicans*, or *Rhizopus arrhizus* compared to the levels when NK-92 cells are incubated alone. Different to primary human NK cells, no increase of perforin levels in the supernatant was observed when the fungi were added to NK-92 cells. Our in vitro data demonstrated that the NK-92 cell line could be a feasible tool for antifungal immunotherapy, but data of animal models are warranted prior to clinical trials.

## 1. Introduction

Pediatric and adult patients undergoing allogeneic hematopoietic stem cell transplantation (HSCT) are at an increased risk for invasive fungal disease (IFD), which is still associated with high morbidity and mortality [1,2]. The incidence of IFDs in allogeneic HSCT recipients is approximately 15% [3,4]. In addition, despite the availability of new and potent antifungal compounds, such as broad-spectrum triazoles or echinocandins, the mortality of invasive fungal infections remains unacceptably high, and studies report on mortality rates between 50% and over 80% [3,5]. *Aspergillus* spp., *Candida* spp., as well as mucormycetes are the most important pathogens causing these infections, which may occur despite potent antifungal prophylaxis [1,2]. It has become clear that the antifungal host response plays an important role determining the infection risk and outcome of IFD. For example, severe and prolonged neutropenia is the most important risk factor of IFD, and the functional activity of neutrophils, which is suppressed after HSCT, has been shown to be a prognostic factor in invasive aspergillosis [6,7]. Similarly, a T_H_1 response and higher numbers of natural killer (NK) cells are associated with better outcome in IFD [1,6,8]. In order to restore the impaired host immunity after allogeneic HSCT to prevent or to treat IFD, there is growing interest in adoptive immunotherapy transferring effector cells to the patient. Importantly, negative results of studies evaluating the clinical benefit of granulocyte transfusions are most likely due to insufficient number and administrations of granulocytes [9]. The generation of antifungal-specific T_H_1 cells, which have limited cross-reactivity, is still laborious and time consuming [10,11].

It has been demonstrated that primary NK cells are able to kill tumor cells [12] and are active against virus-infected cells, bacteria, and a variety of fungal pathogens (reviewed in [13]). In vitro data are supported by animal studies that show, for example, that the depletion of NK cells in *Aspergillus fumigatus-*infected mice resulted in lower lung levels of interferon (IFN)-γ and increased fungal load [14]. In the clinical setting, the number of NK cells is associated with the risk and outcome of invasive aspergillosis in HSCT patients [6,15]. 

Various studies have demonstrated that the benefit of an immunotherapeutic approach depends on a number of prerequisites, e.g., that the cellular product has to be rapidly accessible and consists of high cell counts, both of which are a limiting factor when using primary human NK cells [9,16]. Therefore, NK cell lines such as NK-92 may be considered as an ideal source for cell-based immunotherapy, as NK-92 is standardized, well-characterized, and can be cryopreserved in a GMP-compliant master cell bank, from which it can be easily and reproducibly expanded [17]. The cell line exhibits cytotoxicity against a broad spectrum of tumor targets in vitro such as various leukemia, lymphoma, and myeloma cell lines as well as primary leukemic blasts [12,18,19,20], and clinical studies report on excellent safety and tolerability of adoptively transferred NK-92 cells [21,22,23]. As it is unclear to date whether and to what extent NK-92 cells are able to damage medically important fungi, we analyzed the effect of NK-92 cell co-incubation with different species of *Aspergillus*, *Candida*, mucormycetes, and *Fusarium.*

## 2. Materials and Methods 

### 2.1. NK-92 Cell Cultivation

Cells of the human NK cell line NK-92 (kindly provided by Torsten Tonn, Institute for Transfusion Medicine and German Red Cross Blood Donation Service North-East, Dresden, Germany) were cultivated in RPMI medium 1640 (1X) + GlutaMAX-I medium (Gibco, Paisley, UK) supplemented with 100 U/mL recombinant human interleukin (rhIL)-2 (Novartis, Basel, Switzerland) and 5% human frozen plasma (HFP; German Red Cross Blood Donor Service Baden-Wuerttemberg—Hessen, Frankfurt, Germany).

### 2.2. Assessment of the Antifungal Activity of NK-92 Cells

The antifungal activity of NK-92 cells was tested with different species of *Aspergillus* (*n* = 4), *Candida* (*n* = 3), mucormycetes (*n* = 6), and *Fusarium* (*n* = 4). It was analyzed using a colorimetric assay using XTT (2,3-bis[2-methoxy-4-nitro-5-sulphenyl]2H-tetrazolium-5-carboxyanilide) sodium salt (Sigma-Aldrich, Steinheim, Germany) plus coenzyme Q_0_ (2,3-dimethoxy-5methyl-1,4-benzoquinone; Sigma-Aldrich), as described previously [24]. Briefly, NK-92 cells were co-incubated with the fungus for up to 6 h at different effector-to-target (E:T) ratios (5:1, 10:1 and 20:1) on the basis of the number of conidia or yeasts used for the formation of hyphae RPMI medium 1640 (1×) + GlutaMAX-I medium (Gibco) supplemented with 100 U/mL rhIL-2 (Novartis). Fungal hyphae incubated alone served as control. NK-92 cells were lysed with sterile distilled water, and hyphae were incubated in an XTT solution (0.25 mg/mL) supplemented with 40 µg/mL coenzyme Q_0_ at 37 °C for 1 h. The absorbance of the supernatant was assessed spectrophotometrically at 450 nm using a 690 nm reference. Hyphal damage was calculated as follows: hyphal damage [%] = (1–X/C) × 100, where X is the absorbance of experimental wells and C is the absorbance of control wells with hyphae only.

### 2.3. Preparation of the Fungi

*Aspergillus fumigatus* (strain AF4215) and *Aspergillus terreus* (clinical isolate identified by sequencing [25], provided by Cornelia Lass-Flörl, Medical University of Innsbruck, Innsbruck, Austria); *Aspergillus* niger complex (*Aspergillus niger*, clinical isolate identified by MALDI-TOF, University Hospital Frankfurt, Germany); and *Aspergillus flavus* (clinical isolate identified by MALDI-TOF, University Hospital Frankfurt, Germany) were grown on Sabouraud glucose agar plates (BD Bioscience, San Jose, CA, USA) at 37 °C for 2–3 days. 

*Lichtheimia ramosa*, *Lichtheimia corymbifera*, *Rhizopus microsporus*, *Rhizomucor pusillus*, *Cunninghamella blakesleeana*, and *Rhizopus arrhizus* (all clinical isolates identified by sequencing, provided by Cornelia Lass-Flörl, Medical University of Innsbruck, Innsbruck, Austria) were grown on cornmeal–glucose–sucrose–yeast extract agar plates at 30 °C for 3 to 4 days. 

*Fusarium oxysporum*, *Fusarium solani*, *Fusarium proliferatum*, and *Fusarium verticillioides* (all clinical isolates identified by sequencing, provided by Cornelia Lass-Flörl, Medical University of Innsbruck, Innsbruck, Austria) were grown on potato-dextrose agar plates (PDA, BD; Dextrose PanReak AppliChem, Darmstadt, Germany) at room temperature for up to 2 weeks.

*Candida albicans* (ATCC 90028), *Candida tropicalis* (ATCC 750), and *Candida dubliniensis* (clinical isolate identified by MALDI-TOF and sequencing, University Hospital Frankfurt, Germany) were grown on Sabouraud glucose agar plates (BD Bioscience) at 37 °C for 2 to 3 days.

Conidia and yeasts were harvested by gently scraping the surface of the plates, then washed in DPBS (Gibco) and filtered through a sterile 40 µm cell strainer. The number of the conidia and yeasts was estimated in a Neubauer chamber (LO–Laboroptik, Friedrichsdorf, Germany). Resting conidia and yeasts were used immediately for the experiments. 

Hyphae of the different fungi were prepared as described previously [24,26,27]. In brief, 200 µL of conidia suspension of *Aspergillus* spp. or of mucormycetes (5 × 10^4^ conidia per mL) were plated in 96-well flat-bottom cell culture plates (Nunc, Langenselbold, Germany) and incubated in yeast nitrogen base (YNB; Sigma-Aldrich, Taufkirchen, Germany) medium at 37 °C for 17 h to allow formation of hyphae. On the basis of previous results, hyphae of *Candida* spp. were prepared by plating yeast suspension (200 µL, 2.5 × 10^5^ per mL) in RPMI medium containing 5% human serum (Biochrom AG, Berlin, Germany) in 96-well flat-bottom cell culture plates (Nunc) at 37 °C for 6 h. Hyphae of *Fusarium* spp. were generated by plating 1 × 10^5^ conidia per milliliter for 22 h (*F. oxysporum*, *F. solani*) or for 24 h (*F. proliferatum*, *F. verticillioides*) at 37 °C.

### 2.4. Assessment of the Degranulation of NK-92 Cells

Degranulation of NK-92 cells was evaluated as previously described with some modifications [28]. In brief, a total of 5 × 10^5^ NK-92 cells were incubated in 500 µL RPMI medium 1640 (1×) + GlutaMAX-I medium (Gibco) in the presence or absence of *A. fumigatus* or *R. arrhizus* hyphae. After adding 5 µL anti-CD107a-PE-Cy7 and 0.3 µL BD GolgiStop (both BD Biosciences) and incubating at 37 °C for 4 h, we assessed degranulation by flow cytometry (FACSCantoII, Becton Dickinson, San Jose, CA, USA) using the following antibodies: anti-CD3-APC-Cy7, anti-CD45-FITC, and anti-CD56-APC (all BD Biosciences), and 7-amino-actinomycin-D (Beckman Coulter, Krefeld, Germany). 

### 2.5. Assessment of the Concentration of Soluble Molecules in the Supernatant

Supernatants were collected after NK-92 cells had been incubated for 2, 4, and 6 h with or without the fungus. Levels of interferon (IFN)-γ, which is released by NK cells and modulates the immune system (limit of detection, 0.99 pg/mL), and perforin, which mediates direct cytotoxicity (limit of detection, 11.8 pg/mL), were analyzed by means of commercially available ELISA (Invitrogen, Thermo Fisher Scientific, Carlsbad, CA, USA) according to the manufacturer’s instructions. Each experiment was performed in duplicate.

### 2.6. Statistical Analyses

Data were analyzed using GraphPad Prism (version 5.04; GraphPad Software, La Jolla, CA, USA). One-way ANOVA was used to compare multiple groups of datasets. A two-sided *p*-value of less than 0.05 was considered to be statistically significant. 

## 3. Results

### 3.1. NK-92 Cells Damaged Hyphae of a Variety of Medically Important Fungi

**Figure 1:**
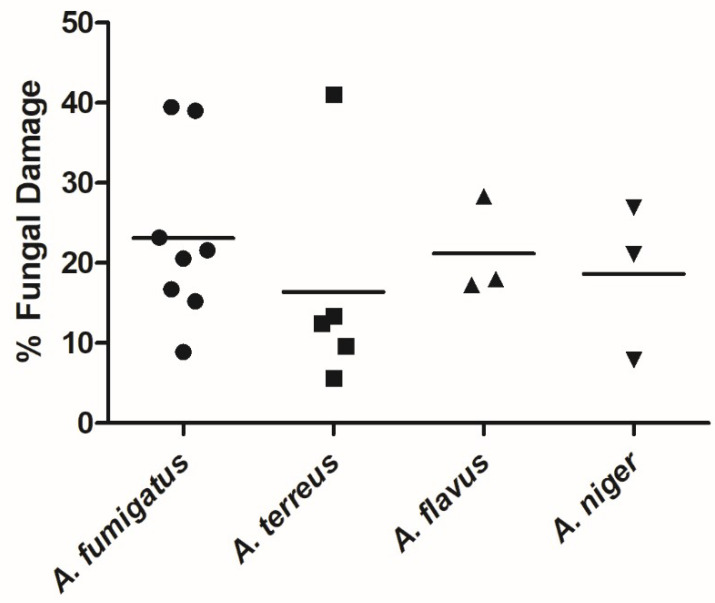
Hyphae of *A. fumigatus*, *A. terreus*, *A. flavus*, and *A. niger* were damaged by natural killer (NK)-92 cells. Hyphae of different species of *Aspergillus* were co-incubated with NK-92 cells for 6 h in the presence of 100 U/mL recombinant human interleukin (rhIL)-2. Hyphal damage was assessed by means of XTT (2,3-bis[2-methoxy-4-nitro-5-sulphenyl]2H-tetrazolium-5-carboxyanilide) assay. Horizontal bars represent the mean.

Our data show that NK-92 cells damaged all *Aspergillus* spp. tested, namely, *A. fumigatus*, *A. terreus*, *A. flavus*, and *A. niger* (Figure 1). Specifically, after 6 h of co-incubation at an E:T ratio of 20:1, NK-92 cells damaged [(mean ± standard error of the mean (SEM)] 23.1% ± 3.9% of *A. fumigatus* hyphae, 16.4% ± 6.3% of *A. terreus* hyphae, 21.2% ± 3.6% of *A. flavus* hyphae, and 18.7% ± 5.6% of *A. niger* complex hyphae. There was no significant difference of the extent of damage between the different species.

**Figure 2:**
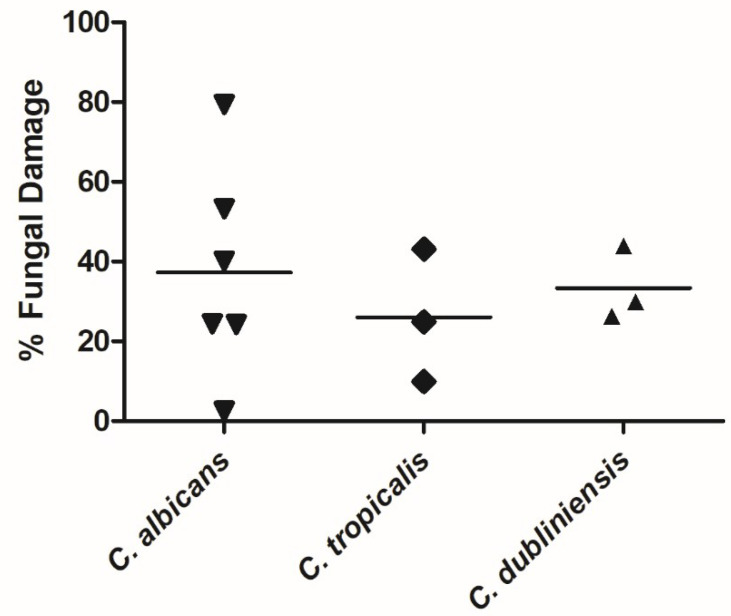
Hyphae of *C. albicans*, *C. tropicalis*, and *C. dubliniensis* were damaged by NK-92 cells. Hyphae of different species of *Candida* were co-incubated with NK-92 cells for 6 hours in the presence of 100 U/mL rhIL-2. Hyphal damage was assessed by means of the XTT assay. Horizontal bars represent the mean.

NK-92 cells also exhibited antifungal activity against all three *Candida* species tested, and damaged after 6 hours (mean ± SEM) 37.3% ± 10.9%, 26.0% ± 9.6%, and 33.3% ± 5.4% of *C. albicans*, *C. tropicalis,* and *C. dubliniensis* hyphae, respectively (Figure 2). No significant differences in fungal damage were seen between the different species.

**Figure 3:**
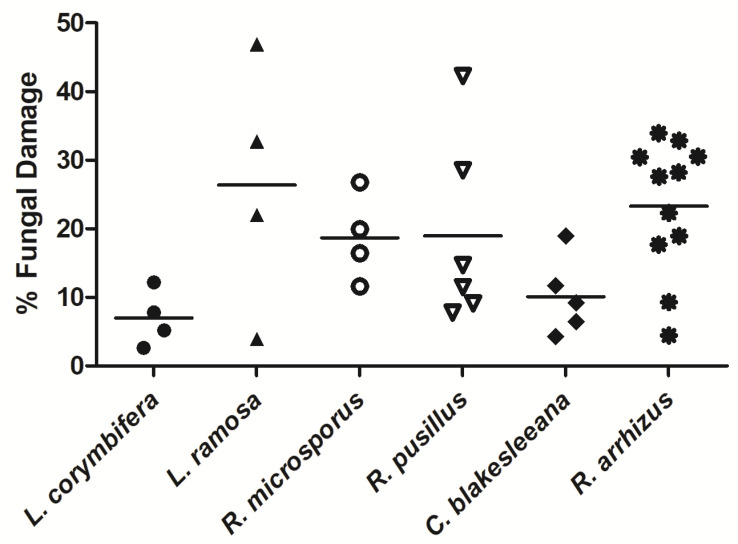
NK-92 cell-mediated hyphal damage of various mucormycetes. Hyphae of *L. corymbifera, L. ramosa*, *R. microsporus*, *R. pusillus, C. blakesleeana*, and *R. arrhizus* were co-incubated with NK-92 cells for 6 hours in the presence of 100 U/mL rhIL-2. Hyphal damage was assessed by means of the XTT assay. Horizontal bars represent the mean.

NK-92 cells exhibited hyphal damage on all mucormycetes tested (Figure 3). The mean hyphal damage of mucormycetes varied between 7.0% ± 2.0% (mean ± SEM; *L. corymbifera*) and 26.4% ± 9.1% (*L. ramosa*). The extent of damage by NK-92 cells was comparable for *L. ramosa*, *R. microsporus*, *R. pusillus*, and *R. arrhizus* (all between 18.7% ± 3.2% and 26.4% ± 9.1%), whereas the damage of *L. corymbifera* and *C. blakesleeana* was lower (7.0% ± 2.0% and 10.1% ± 2.5%, respectively). No significant differences of hyphal damage were detected between the various mucormycetes.

**Figure 4:**
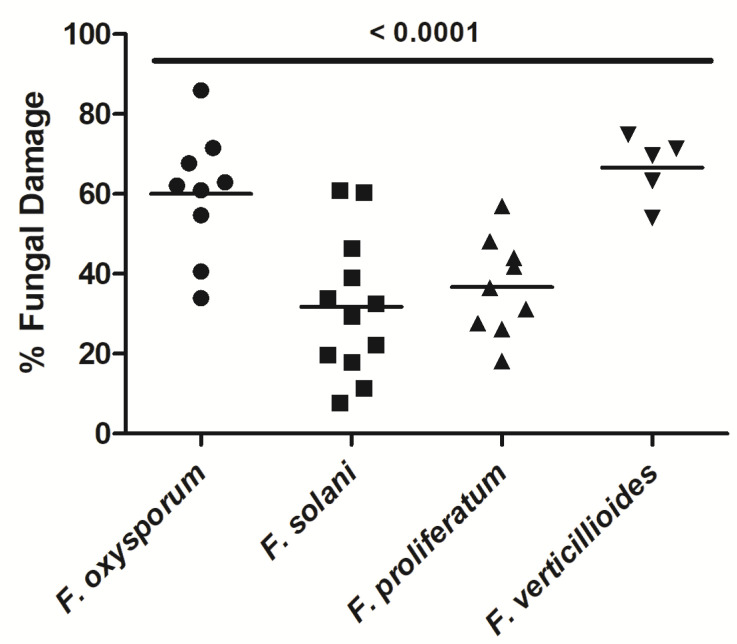
Hyphae of different species of *Fusarium* were damaged by NK-92 cells. Hyphae of *F. oxysporum, F. solani, F. proliferatum,* and *F. verticillioides* were co-incubated with NK-92 cells for 6 h in the presence of 100 U/mL rhIL-2. Hyphal damage was assessed by means of the XTT assay. Horizontal bars represent the mean.

Although NK-92 cells damaged all *Fusarium* species tested, the extent of mean damage was significantly different (*P* < 0.0001) and ranged from 31.8% ± 5.1% (mean ± SEM; *F. solani*) to up to 66.7% ± 3.7% (*F. verticillioides*) (Figure 4). The extent of damage of *F. oxysporum* and *F. verticillioides* hyphae by NK-92 cells was significantly higher than that of *F. solani*. 

### 3.2. The Extent of Fungal Damage of Aspergillus fumigatus, Candida flbicans, and Rhizopus frrhizus Depended on the Co-Incubation Period and Effector/Target Ratio

**Figure 5:**
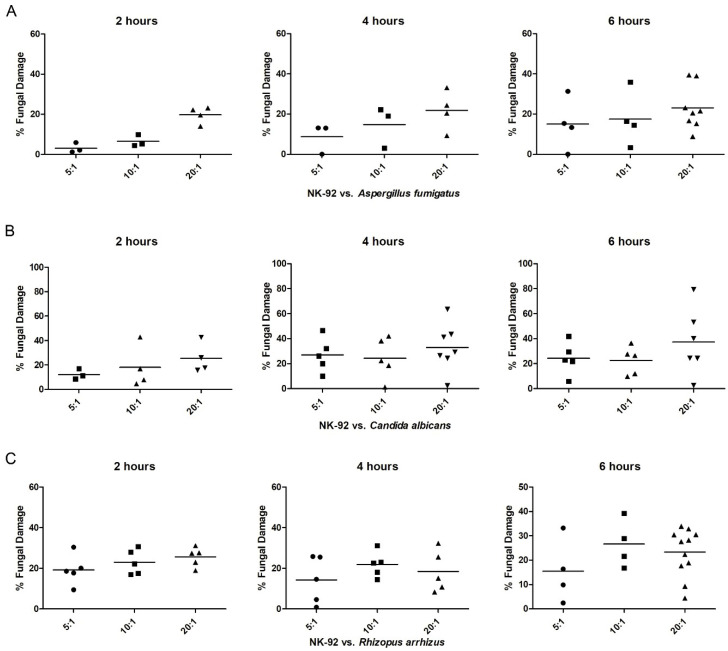
Damage of hyphae of *A. fumigatus* (**A**)*, C. albicans* (**B**)*,* or *R. arrhizus* (**C**) by NK-92 cells. Hyphae of the specific fungi were co-incubated with NK-92 cells at different effector-to-target (E:T) ratios (5:1, 10:1, and 20:1) in the presence of 100 U/mL rhIL-2 for 2 (left), 4 (center), and 6 hours (right). Fungal damage was assessed by means of the XTT assay. Horizontal bars represent the mean.

Analyzing the effect of NK-92 cells on *A. fumigatus* demonstrated that increasing E:T ratios resulted in increased fungal damage. When NK-92 cells and *A. fumigatus* hyphae were co-incubated for 2 hours, a statistically not significant difference was seen between the fungal damage using E:T ratios of 5:1 and 10:1, respectively, and by using an E:T ratio of 20:1 (mean ± SEM: 0.9% ± 2.6% at 5:1, 6.5% ± 1.7% at 10:1, and 19.8% ± 2.1% at 20:1) (Figure 5A). Similarly, when comparing the co-incubation periods of 2, 4, and 6 hours, the hyphal damage increased for a specific E:T ratio over time. Compared to an E:T ratio of 20:1, this increase was more pronounced for an E:T ratio of 5:1 and 10:1, but did not reach statistical significance.

Co-incubation of NK-92 cells with *C. albicans* for 2 h with increasing E:T ratios resulted in increasing mean hyphal damage (mean ± SEM) 12.2% ± 2.5% at an E:T ratio of 5:1, 18.0% ± 8.7% at 10:1, and 25.4% ± 6.1% at 20:1 (Figure 5B), which, however, was not statistically different. No major differences were seen for the hyphal damage of the fungus after 4 and 6 hours of co-incubation, independently from the E:T ratio used.

The mean damage of *R. arrhizus* by NK-92 cells varied between 14.3% (E:T ratio 5:1, 4 h co-incubation) and 26.6% (E:T ratio 10:1, 6 h co-incubation), and did not demonstrate a specific pattern with increasing fungal damage in relation to increasing E:T ratios or longer periods of co-incubation (Figure 5C).

### 3.3. Assessment of Perforin Levels in the Supernatant and of the Degranulation Marker CD107a on the Surface of NK-92 Cells

**Figure 6:**
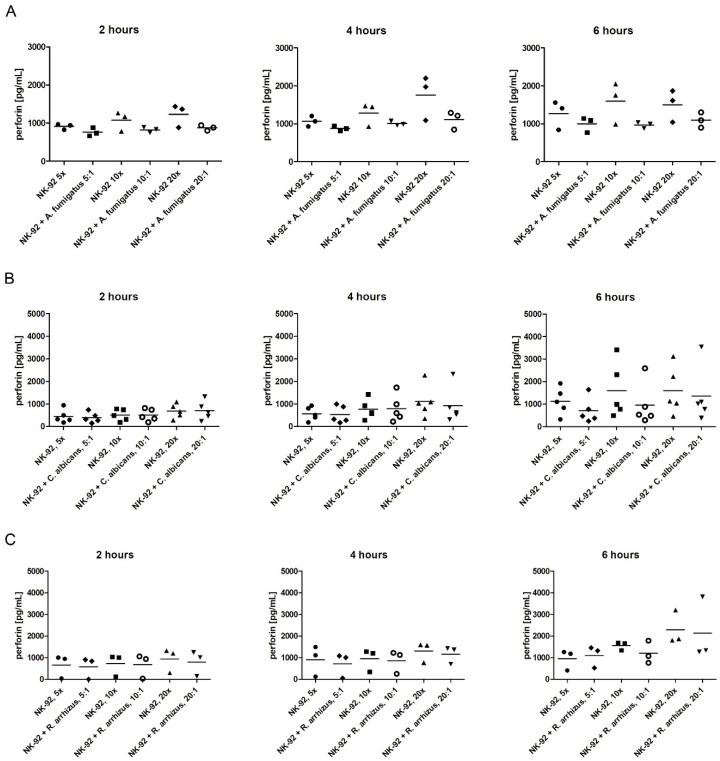
Concentration of perforin in the supernatant when NK-92 cells were incubated alone (empty boxes) or were co-incubated with hyphae of *A. fumigatus* (**A**), *C. albicans* (**B**), or *R. arrhizus* (**C**) (shaded boxes). Perforin levels were assessed by ELISA. Horizontal bars represent the mean.

When adding a fungal pathogen to NK-92 cells, we found measured perforin levels to be lower compared to NK-92 cells incubated alone (Figure 6A-C). Although statistically not significant, this difference was more pronounced after 6 h for all fungi of co-incubation than after 2 or 4 h. For example, co-incubation of NK-92 cells for 2 h with *A. fumigatus* at E:T ratios of 5:1, 10:1, and 20:1, resulted in perforin levels of (mean ± SEM) 765.2 pg/mL ± 64.0 pg/mL, 827.3 pg/mL ± 43.3 pg/mL, and 877.6 pg/mL ± 40.5 pg/mL, respectively, whereas perforin levels of NK-92 cells incubated alone were 913.9 pg/mL ± 43.9 pg/mL, 1076.0 pg/mL ± 147.5 pg/mL, and 1230.0 pg/mL ± 173.0 pg/mL, respectively. Co-incubation for 6 h with *A. fumigatus* at E:T ratios of 5:1, 10:1, and 20:1 led to perforin levels of 1002.9 pg/mL ± 115.4 pg/mL, 970.5 pg/mL ± 46.6 pg/mL, and 1098.2 pg/mL ± 116.0 pg/mL, respectively, compared to corresponding perforin levels of 1270.2 pg/mL ± 219.5 pg/mL, 1597.0 pg/mL ± 315.4 pg/mL, and 1507.3 pg/mL ± 242.8 pg/mL, respectively, when NK-92 cells were incubated alone (Figure 6A). A similar pattern was seen when NK-92 cells were co-incubated with *R. arrhizus*, whereas *C. albicans* altered the perforin levels not before 6 h of co-incubation (Figure 6B, C).

In contrast to the slightly decreased perforin levels, there was an upregulation of the surface expression of the degranulation marker CD107a upon co-incubation of NK-92 cells with hyphae of *A. fumigatus* and *R. arrhizus* (Figure S1).

### 3.4. Assessment of IFN-γ Levels in the Supernatant

**Figure 7:**
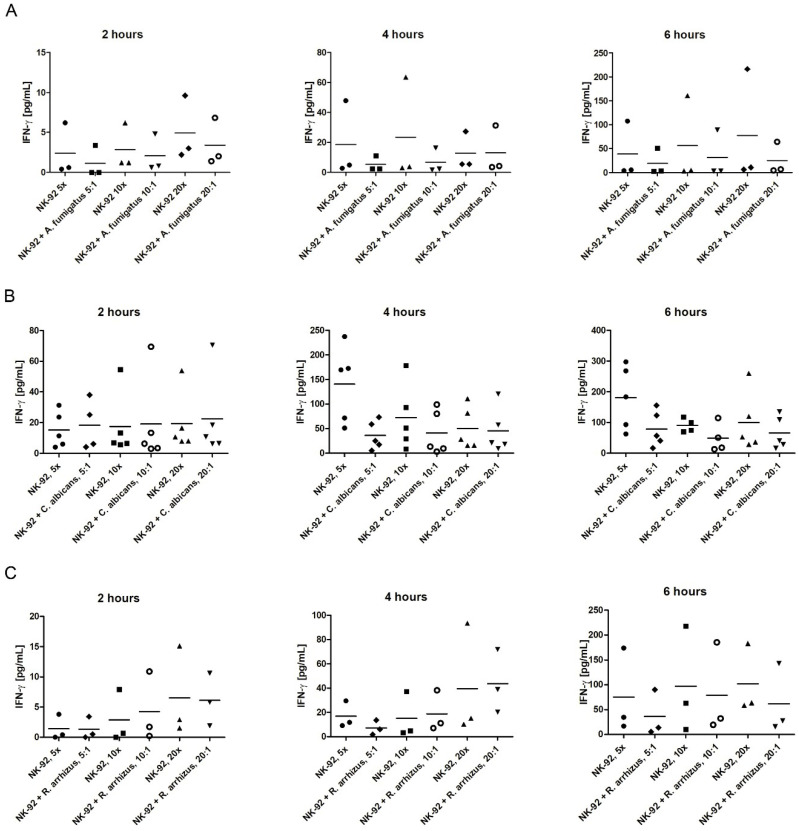
Concentration of interferon (IFN)-γ in the supernatant when NK-92 cells were incubated alone (empty boxes) or were co-incubated with hyphae of *A. fumigatus* (**A**), *C. albicans* (**B**), or *R. arrhizus* (**C**) (shaded boxes). IFN-γ levels were assessed by ELISA. Horizontal bars represent the mean.

When NK-92 cells were co-incubated with *A. fumigatus,* IFN-γ levels in the supernatant were lower at all time points analyzed compared to those of NK-92 cells incubated alone, although these differences were statistically not significant (Figure 7A). For example, co-incubation of NK-92 cells and the fungal pathogen for 6 h at E:T ratios of 5:1, 10:1, and 20:1 resulted in IFN-γ levels of 19.2 pg/mL ± 15.8 pg/mL, 31.7 pg/mL ± 28.6 pg/mL, and 25.0 pg/mL ± 19.5 pg/mL, respectively, whereas corresponding IFN-γ levels of NK-92 cells incubated alone were 39.0 pg/mL ± 34.2 pg/mL, 56.4 pg/mL ± 52.2 pg/mL, and 77.9 pg/mL ± 69.3 pg/mL, respectively. The decrease in IFN-γ levels was also observed after 4 and 6 h of co-incubation of NK-92 cells with *C. albicans* (Figure 7B), and after 6 h of co-incubation of NK-92 cells with *R. arrhizus* (Figure 7C).

## 4. Discussion

Despite the availability of new and potent antifungal compounds such as broad- spectrum triazoles or the new class of echinocandins, morbidity and mortality of IFD remains unacceptably high, particularly in allogeneic HSCT recipients. For example, a single-center retrospective study including 404 patients undergoing HSCT reported a 1-year cumulative incidence of IFD of 11% despite mold-active antifungal prophylaxis [1]. The non-relapse mortality was 16% in patients who did not develop IFD, but reached 40% in those in whom IFD occurred. As therapy-induced alterations of the number and function of phagocytes, NK cells or the cellular immune system are known to increase the risk for IFD and are associated with poor outcome [1,6,8]; there is growing interest in adoptive immunotherapeutic strategies for restoring immunity to prevent or to treat IFD. Unfortunately, to date, clinical studies have failed to demonstrate the benefit of immunotherapeutic approaches in infectious complications. This is due, at least in part, to the fact that effector cells are often available in limited numbers only and are not timely accessible, as the generation of the immunotherapeutic cellular product such as fungal-specific T cells requires donor cells and is laborious and time-consuming [9,10,11]. Therefore, standardized and well-characterized cell lines would be an ideal source for adoptive immunotherapy in patients with IFD.

Although it has been shown that NK-92 cells are able to kill tumor cells [12] and are active against virus-infected cells [29,30,31], the ability of NK-92 cells to damage fungi has not been investigated to date. Therefore, one of our main findings was the observation that NK-92 cells exhibit fungal damage on all medically important fungi tested, namely, on different species of *Aspergillus*, *Candida*, mucormycetes, and *Fusarium*. The extent of fungal damage significantly differed between the species of *Fusarium*, whereas it was comparable across different species of *Aspergillus,* mucormycetes, and *Candida*. Results of experiments performed in the identical setting as the present studies demonstrate not only a comparable anti-*Aspergillus* and anti-mucormycete activity of NK-92 cells and primary human NK cells, but also an anti-*Aspergillus* activity in the range of that seen in this setting by pharmacological dosages of the antifungal compounds caspofungin or voriconazole [21,23,24,32]. Studies in freshly isolated NK cells and polymorphonuclear leukocytes have suggested that the extent of fungal damage not only depends on the fungal species, but also on fungal growth characteristics, with higher damage in lower biomass [27,33]. For the clinical setting, these data imply that adoptive immunotherapy using NK-92 cells should start as early as possible with a maximum tolerated number of NK-92 cells. Clinical studies evaluating NK-92 cells as immunotherapy against a malignancy reported that dosages up to 10^10^ cells per square meter were well tolerated with no or minimal toxicities [21,22,23]. In addition, similar to the fact that most patients with proven or probable IFD receive one or more antifungal drugs over weeks and months, it might be necessary in the clinical situation to repeat NK-92 immunotherapy multiple times. To this end, the rapid availability of sufficient number of cells and the possibility to administer the cells multiple times over weeks favors NK-92 cells compared to primary human NK cells for antifungal immunotherapy, in particular as the antifungal activity in vitro is comparable.

*A. fumigatus* is damaged by purified human perforin, and blocking perforin-mediated toxicity by concanamycin A significantly decreases fungal damage by IL-2 pre-stimulated human NK cells, which clearly indicates that NK cell-derived perforin plays an important role in the antifungal activity of NK cells [24]. Whereas previous studies with primary human NK cells demonstrated a direct correlation of the upregulation of the degranulation marker CD107a and increasing concentrations of perforin in the supernatant [26,34], we found a slight, but not significant decrease of the perforin levels in the supernatant of NK-92 cells. Perforin reduces the metabolic activity of hyphae of *A. fumigatus* [24], *R. arrhizus* [26], and of *C. albicans* [35], inhibits hyphal elongation of *C. albicans* [35], and growth of *Cryptococcus neoformans* [36]. However, the exact mechanism of perforin-mediated damage of fungal cells is not clear to date. Previous data have shown that in human NK cells, *A. fumigatus* downregulates mRNA levels of perforin, but increases both intracellular concentration and the release of the molecule, resulting in higher extracellular perforin concentrations [37]. This is in contrast to the present results, which demonstrate that co-incubation of NK-92 cells with *A. fumigatus*, *C. albicans*, or *R. arrhizus* did not lead to an increase of perforin levels in the supernatant. The fact that increased fungal damage was observed although the levels of perforin in the supernatant did not increase may be explained by the fact that other NK-92-derived molecules exhibit direct antifungal activity or that there is a consumption of the molecule while damaging the fungi. We did not assess the levels of granulysin and granzyme B in the supernatant, but it is important to note that granzyme B did not exhibit any effect against *C. albicans* or enhanced the antifungal activity of perforin [34].

Beside the direct NK cell-mediate fungal damage, soluble factors released by NK cells play an important role in the regulation of the antifungal host response. For example, NK cell-derived IFN-γ stimulates the migration of phagocytes and enhances their phagocytic and oxidative killing activity of *A. fumigatus* [38], and is a signature cytokine of protective T_H_1 response [38,39,40]. In a mouse model, it was demonstrated that the depletion of NK cells in *A. fumigatus*-infected animals resulted in lower lung levels of IFN-γ and increased fungal load [14]. The present data show that co-incubation of NK-92 cells with hyphae of *A. fumigatus*, *C. albicans*, or *R. arrhizus* decreased the concentration of IFN-γ in the supernatant compared to that of NK-92 alone; however, when comparing the different fungi, there was no clear pattern with regard to the extent and kinetics. The present results are corroborating a study on human NK cells that demonstrated that incubation of IL-2 stimulated primary NK cells with hyphae of *A. fumigatus* resulted in intracellular accumulation of the IFN-γ protein but reduction of its concentration in the supernatant [37]. The mechanisms causing the extracellular decrease of IFN-γ are unclear to date, but is the focus of our current research, which might help to improve adoptive immunotherapeutic strategies. Another study reported recently that IFN-γ levels in the supernatant of NK cells co-incubated with K562 tumor cells decreased when adding *A. fumigatus* [41]. The authors of this study speculated that this was due to an exhaustion phenotype of the cells. However, whether the reduction of extracellular IFN-γ levels in the presence of fungi ultimately results in an impaired antifungal host response in vivo or are a protecting host effect against hyper-inflammation leading to tissue damage will be a matter of future research.

Despite the potential advantages of the NK-92 cell line such as the rapid availability of high counts of standardized and well-characterized effector cells, a number of potentially important drawbacks for the clinical setting have to be mentioned. First, the NK-92 cell line is derived from a NK cell lymphoma and has the potential risk of uncontrolled proliferation. Therefore, irradiation is mandatory prior to adoptive immunotherapy, but limits the survival and function of the cells. There also are major concerns regarding safety, but an early study performed in Germany, which included 15 patients including children (patients’ ages were 9-71 years) with advanced, treatment-resistant malignancies who received two infusions of NK-92 cells did not report a negative effect of NK-92 cells on blood cell counts or renal and hepatic function [21]. Similarly, in an ongoing phase I clinical trial [CAR2BRAIN (NCT03383978, clinicaltrials.gov (accessed on 1 February 2021))], which has undergone rigorous evaluation by regulatory authorities as well by an ethical committee, NK-92 cells are administered intracranially as a therapeutic approach in patients with recurrent or refractory ErbB2-positive glioblastoma. In the first cohort of nine patients treated until the end of 2019, no adverse events related to the NK-92 lymphoma cells occurred (personal communication by Michael Burger). In addition, NK-92 cells depend on IL-2, and repeated IL-2 injections raise concerns regarding toxicity [18,42]. Interestingly, a preclinical study reported on the transduction of NK-92 cells with lentiviral vectors encoding human IL-15, which resulted in a predominantly intracellular expression of the cytokine, and proliferation and cytotoxicity of the cells in the absence of IL-2 [43]. It is also important to mention that other cell lines such as the KHYG-1 cell line, which is derived from NK leukemia, or the cell line NKL [44], which is biologically and functionally very similar to primary NK cells, may be superior compared to NK-92 cells for adoptive immunotherapy of IFD, but the antimicrobial activity of these cell lines is unclear to date and has to be evaluated prior to clinical use. 

In conclusion, our data demonstrate that NK-92 cells exhibit antifungal activity against a broad range of fungal pathogens such as *Aspergillus* spp., *Candida* spp., mucormycetes, and *Fusarium* spp. and may be suitable as a standardized and rapidly available cell product for adoptive immunotherapy of IFD in allogeneic HSCT patients, but data of animal models are warranted prior to clinical trials. 

## Data Availability

The data that support the findings of this study are available from the corresponding author upon reasonable request.

## Supplementary Materials

The following are available online at https://www.mdpi.com/2309-608X/7/2/144/s1, Figure S1: Hyphae of A. fumigatus (left) and R. arrhizus (right) induce degranulation of NK-92 cells.
