# Supplementary material for: Natural Killer Cell Line NK-92-Mediated Damage of Medically Important Fungi
Source: J Fungi (Basel). 2021 Feb 17;7(2):144. doi: 10.3390/jof7020144 (PMC7922546; doi:10.3390/jof7020144)
Supplement: Supplementary file 1 [file jof-07-00144-s001.pdf]

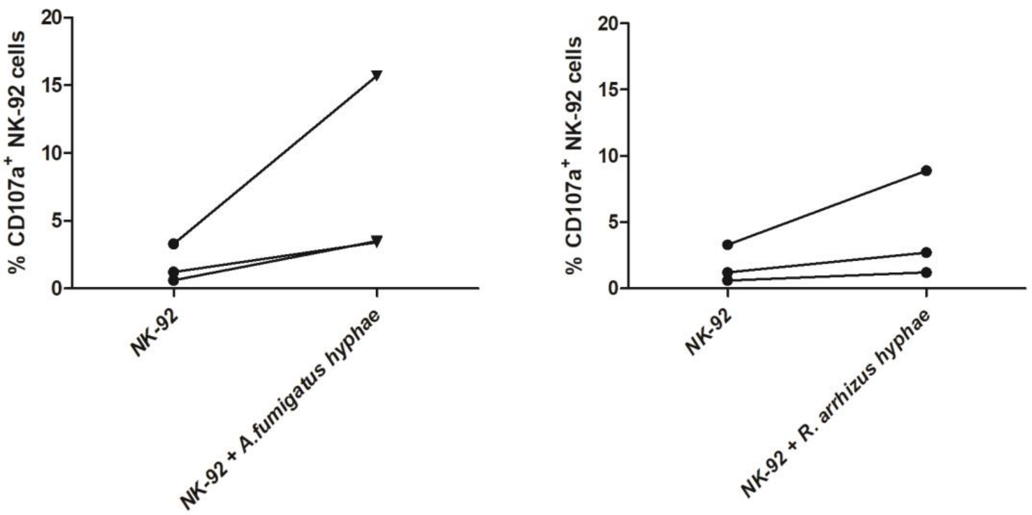

Supplemental Figure S1: Hyphae of *A. fumigatus* (left) and *R. arrhizus* (right) induce degranulation of NK-92 cells. NK-92 cells were incubated either alone or co-incubated with the fungi for 4 hours. The degranulation marker CD107a was assessed by flow cytometry.
